# APOE4-Expressing Astrocytes Exhibit Parkinson’s Disease–Related Pathology

**DOI:** 10.1007/s12035-026-05996-5

**Published:** 2026-06-11

**Authors:** Lior Nechushtai, Anna Popov, Roni Haklai, Daniel M. Michaelson, Dan Frenkel, Ronit Pinkas-Kramarski

**Affiliations:** School of Neurobiology, Biochemistry and Biophysics, Department of Neurobiology, Tel-Aviv University, Ramat-Aviv, 69978 Israel

**Keywords:** Parkinson’s disease (PD), Alpha-synuclein, Apolipoprotein E4 (apoE4), Autophagy

## Abstract

Parkinson’s disease (PD) is characterized by motor symptoms that are mainly attributed to the progressive loss of dopaminergic neurons of the substantia nigra (SN). It is also characterized by abnormal inclusion vesicles, termed Lewy bodies (LBs), enriched with α-synuclein aggregates that may induce inflammation and neurotoxicity. The possibility that factors involved in other neurodegenerative diseases also affect PD-related pathologies, such as α-synuclein uptake, was examined. The *apoe4* allele is a major genetic risk factor for Alzheimer’s disease (AD) and has also been suggested to be involved in PD. Here, we examined the effects of APOE isoform expression on α-synuclein uptake and autophagy in astrocytes expressing the *apoe3* or *apoe4* alleles. Using multiple autophagy manipulations (EBSS, chloroquine, and rapamycin treatments), we found that α-synuclein uptake and autophagy readouts differ between *APOE3* and *APOE4* astrocytes, supporting a functional link between autophagy status and α-synuclein levels. Astrocytes expressing *APOE4* exhibit reduced uptake of α-synuclein and reduced autophagy. Moreover, α-synuclein treatment inhibits autophagy mainly in *APOE3*-expressing cells. Additional experiments showed that the autophagy inhibitor chloroquine reduced α-synuclein uptake in *APOE3* astrocytes but not in *APOE4* astrocytes, while the autophagy enhancer rapamycin increased α-synuclein uptake in *APOE4*-expressing astrocytes. In addition, we found that Toll-like receptor 2 (TLR2) levels are elevated at both the mRNA and protein levels in *APOE4*-expressing astrocytes, whereas α-synuclein increased only TLR2 mRNA levels in *APOE3*-expressing astrocytes. Using the neurotoxin 1-methyl-4-phenylpyridinium (MPP^+^), we found that it affects cell growth in both *APOE3* and *APOE4*-expressing astrocytes. MPP^+^ treatment also reduced autophagy which was partially corrected by rapamycin. Taken together, these findings show that in astrocytes, *APOE4* impairs α-synuclein uptake, which was emended by rapamycin and α-synuclein inhibits autophagy mainly in *APOE3*. These findings suggest that autophagy-targeting strategies can modulate astrocyte α-synuclein uptake; however, given the observed reductions in astrocyte cell number following rapamycin treatment, further optimization or examination of alternative autophagy modulators is needed.

**Supplementary Information:**

The online version contains supplementary material available at 10.1007/s12035-026-05996-5.

## Introduction

Parkinson’s disease (PD), the second most common neurodegenerative disease, is characterized by the presence of Lewy bodies (LBs) that contain many proteins, including α-synuclein aggregates, at the substantia nigra (SN), leading to degeneration of resident dopaminergic neurons. Dopamine metabolism has been identified as a possible source of oxidative stress, contributing to the enhanced degeneration of dopaminergic neurons in PD [1]. The factors contributing to these pathologies remain elusive [2], and it is possible that factors involved in the generation of other neurodegenerative diseases also affect α-synuclein accumulation and dopamine metabolism, thus promoting PD pathogenesis. The *apoe4* allele, which is a major genetic risk factor for Alzheimer’s disease (AD) and was previously shown to affect amyloid-beta aggregation and accumulation in senile plaques, might also affect pathological mechanism involved in PD [3].

The gene encodes a lipid carrier protein that exists in three isoforms (*APOE2*, *APOE3*, and *APOE4*). It is the most abundant apolipoprotein in the brain and is produced and secreted mainly by astrocytes [4–6]. The detrimental effects of *APOE4* in AD include compromised cellular functions of astrocytes and microglia, which also play an essential role in PD pathology [7, 8], e.g., decreased uptake and removal of protein aggregates, impaired autophagy and endocytosis/phagocytosis, reduced mitochondrial activity, and altered inflammatory response [9–13]. Previously, others and we have shown that autophagy is impaired in *APOE4* astrocytes [10, 13]. Importantly, *APOE4* is also a risk factor in other neurodegenerative disorders, such as multiple sclerosis [14] and Niemann–Pick disease [15]. Although a possible role for *APOE* variants in PD is less documented, studies have suggested that *APOE* is linked to PD pathology, with the *apoe4* allele possessing harmful effects [16–21]. For example, low concentration of APOE was shown to promote α-synuclein aggregation, with *APOE4* exerting the greatest effect; expression of *APOE4* in A53T α-synuclein transgenic mice has led to a higher burden of phosphorylated α-synuclein and gliosis compared to other variants of *APOE*; recombinant *APOE4* was shown to reduce the uptake of α-synuclein by oligodendrocytes; the *apoe4* allele was demonstrated to be associated with increased risk for LBs pathology in AD patients; and PD patients expressing *APOE4* exhibit earlier age of disease onset and the fastest cognitive decline [22]. Although these findings indicate a possible link between *APOE4* and PD, the mechanisms mediating the effects of *APOE4* on α-synuclein aggregation/removal and overall PD progression are unclear. In addition, the effects of *APOE4* on glial cell functions in the context of PD are still unknown.

In the present study, the involvement of *APOE4* in several cellular changes related to PD was investigated. We found that following exposure to α-synuclein, *APOE4*-expressing astrocytes demonstrate decreased uptake of α-synuclein compared to *APOE3* astrocytes. Furthermore, *APOE3* cells demonstrated decreased autophagy following α-synuclein treatment, compared to *APOE4* cells. MPP^+^ treatment reduced autophagy in both *APOE3* and *APOE4*-expressing astrocytes, which also showed reduced cell proliferation following treatment. Taken together, the current study suggests that *APOE4* impairs the ability of astrocytes to eliminate α-synuclein, which may contribute to PD pathology.

## Materials and Methods

### Materials

Antibodies were obtained from the following sources: polyclonal rabbit anti-LC3B (Sigma-Aldrich, St. Louis, MO; L7543), polyclonal rabbit anti p62/SQSTM1 (MBL International, Woburn, MA, PM045), monoclonal mouse anti-α-synuclein (BD Biosciences, 610787), monoclonal rabbit anti-α-synuclein (phosphoS129) (Abcam [EP1536Y] ab51253), polyclonal rabbit anti TLR2 (BIOSS, bs-1019R-TR), monoclonal mouse anti-actin (MPB; 08691002), polyclonal rabbit anti-DYKDDDDK Epitope Tag (FLAG tag) (BLG, BLG-902401), monoclonal mouse anti-phosphorylated Erk1/2 (Sigma, M8159), monoclonal mouse anti Erk1/2 (Santa Cruz, sc-514302), and monoclonal mouse anti-b-Tubulin (Sigma, T78116). Reagents are as follows: AnaTag™ 5-FITC Protein Labeling Kit (Anaspec, Campus Drive Fremont, CA, 94555, AS-72059), Latex Beads (Sigma), Earle’s Balanced Salts (EBSS) (Sigma-Aldrich, E3024), chloroquine (CQ, Sigma-Aldrich, C6628), rapamycin (Rapa, Cayman Chemical, Ann Arbor, MI, 13346), PitStop2 (Abcam, Cambridge, UK, ab120687), nystatin (Biological Industries, 03–030), methyl-β-cyclodextrin (Sigma-Aldrich, C4555), LysoTracker™ Deep Red (Invitrogen™, L12492), Pam2CysSerLys4 (Pam2CSK4) (InvivoGen, tlr-pm2s-1), Pam3CysSerLys4 (Pam3CSK4) (InvivoGen, tlr-pms), MPP^+^ iodide (Sigma-Aldrich, D048), methylene blue and [3-(4,5-dimethylthiazol-2-yl)−2,5-diphenyl] tetrazolium bromide (MTT) were purchased from Sigma-Aldrich. 

### Cell Cultures

The human *APOE3* and *APOE4*-expressing astrocytes were previously described [23]. Cells were grown in Dulbecco’s Modified Eagle’s Medium/Nutrient Mixture F-12 HAM (Corning, 15–090-CV) supplemented with 10% heat-inactivated fetal bovine serum (FBS, Gibco, 10270106), 1% L-glutamine, 7.5% sodium bicarbonate, and 0.1 mg/ml gentamycin sulfate (Tivan biotech, GEN50-50ML). U87 glioblastoma cells were stably transfected with pCDNA3-*APOE3*-flag or pCDNA3-*APOE4*-flag expression vectors, using Transit-LT1 reagent (mirusbio, MIR 2304) according to the manufacturer’s instruction. Cells were grown in Dulbecco’s Modified Eagle’s Medium (Gibco, 11965092) supplemented with 10% heat-inactivated fetal bovine serum, 100 μg/ml penicillin–streptomycin, and 500 μg/ml geneticin (G418) sulfate (CAS 108321-42−2, Santa Cruz).

Cells were incubated at 37 °C in 5% CO_2_ in air, and the medium was changed every 3–4 days. Cells were passaged when 70% confluent using trypsin (Beit Haemek, L0931-500). Cells were cultured at 30% confluence in growth medium, 1–2 days before each experiment.

### Lysate Preparation and Immunoblotting

Preparation of cell lysates and Western blot analysis were performed as previously described [13]. Immunoreactive bands were detected using enhanced chemiluminescence reagent (Immobilon Crescendo substrate, Millipore). Bands were visualized by Amersham Imager 600 within the linear range, employing the automatic/semiautomatic exposure options, which allows optimal exposure times below saturation to enable accurate quantification (according to manufacturer instructions). Target protein band intensities were quantified using the ImageJ software and normalized to loading controls.

### Expression and Purification of Recombinant α-Synuclein

Recombinant pET-14-b-HIS-α-synuclein was expressed in Bl-21 *E.coli* as previously described [24]. α-Synuclein protein was purified as previously described [24]. The validation of α-synuclein purification was performed by immunoblotting of 50 μg samples of the preparation, and immunoblots were reacted with monoclonal mouse anti-α-synuclein antibodies. Samples consist of over 85% monomeric α-synuclein and about 15% dimers. No higher molecular species were detected.

### Generation of Fluorescent-Tagged α-Synuclein

Recombinant α-synuclein was tagged using the AnaTag™ 5-FITC Protein Labeling Kit (Anaspec, San Jose, CA, USA) according to manufacturer’s instructions. Briefly, 5-FITC was diluted in dimethyl sulfoxide and mixed with 2 mg/ml recombinant α-synuclein for 1 h at room temperature. The conjugate protein was purified by desalting column and stored at −20 °C until used.

### Quantification of Fluorescent α-Synuclein Uptake by Flow Cytometry

Cells were cultured in a 24-well plate and incubated with 0.5 μM 5-FITC-labeled α-synuclein in serum-free medium at 37 °C for 2 h. The cells were then washed with PBS three times, trypsinized, centrifuged at 1500*g* for 5 min, and resuspended in PBS. Mean fluorescence was analyzed in a fluorescence-activated cell sorter (FACScan, Becton Dickinson, Franklin Lakes, NJ) within 30 min.

### Quantification of Fluorescent α-Synuclein Uptake Using Incucyte® Live Cell Imaging

Cells were cultured in a 96-well plate and incubated with 0.5 μM 5-FITC-labeled α-synuclein. The intensity inside the cells was measured using the Incucyte® machine immediately following the addition of 5-FITC α-synuclein, and uptake was measured every 15 min for the duration of 12 h (four fields were taken in the well).

### Extraction of 3KLTg α-Synuclein from 3KL3798 Mouse Brains

Frozen brains from 10-month-old 3KL3798 mice were generously given by the lab of Prof. Dan Frenkel, Tel Aviv University. α-Synuclein was extracted from the mouse brains as described [25], and the TBS (Tris-buffered saline) fraction was used for the experiments. The extracted α-synuclein was analyzed by immunoblot using rabbit anti-α-synuclein (phosphoS129) and mouse anti-α-synuclein antibodies.

### Measurement of Lysosome Acidity by Flow Cytometry

Cells were cultured in a 12-well plate and incubated with 60 nM LysoTracker™ for 30 min at 37 °C. The cells were then washed with PBS three times, trypsinized, centrifuged at 1500*g* for 5 min, and resuspended in PBS. Mean fluorescence was analyzed in a fluorescence-activated cell sorter (FACScan, Becton Dickinson, Franklin Lakes, NJ) within 30 min.

### Transient Transfection with the LC3-EGFP-mRFP Vector

Transient transfection of *APOE3* and *APOE4*-expressing astrocytes with the tandem LC3-EGFP-mRFP (Addgene ptfLC3) was performed using Lipofectamine 2000 reagent (Invitrogen). The cells were treated with or without EBSS and α-synuclein, fixed, and microscopically visualized using Olympus motorized inverted research microscope Model IX81.

### RNA Extraction and qRT-PCR Analysis

Total RNA was extracted from *APOE3* and *APOE4*-expressing astrocytes and 5-month-old *apoe*-target replacement mice, in which the endogenous mouse *apoe* was replaced by either human *apoe*3 or *apoe*4, as previously described [26]. The animals were generously given by the lab of Prof. Daniel Michaelson, Tel Aviv University. Total RNA was extracted using TRizol reagent (Thermo Fisher, 15596026). Purified RNA was reverse transcribed into cDNA using qScript cDNA Synthesis Kit (Quantabio, 95047-100) according to the manufacturer’s instructions. cDNA samples were used for quantitative RT-PCR using the Fast SYBER™ Green Master Mix (Thermo Fisher, 4385612). Fluorescence was measured and the data was analyzed using the Quantbio1 Real-Time PCR system (Rhenium). The primers used were TLR2 forward 5′GCAAACGCTGGTCTGCTCAG3′ and reverse 5′AAGCGTCTCCCTCTATTGTATT3′. GAPDH forward 5′CGGAGTCAACGGATTTGGTC3′ and reverse 5′GAATTTGCCATGGGTGGAAT3′ were used as a control gene for normalization of relative mRNA expression.

### Methylene Blue Viability Assay

Cells were cultured in a 96-well plate and incubated with MPP^+^ as indicated. The cells were fixed with 4% formaldehyde for 2 h, followed by washing with 0.1 M boric acid (pH = 8.5), and were incubated with methylene blue (1% in boric acid) for 20 min at room temperature. Then, the fixed cells were washed with water three times and lysed with 0.1 M HCl. Absorbance was measured at 595 nm.

### MTT Assay

Cells were cultured in a 96-well plate and incubated with MPP^+^ as indicated. Then, cells were incubated with 0.5 mg/ml [3-(4,5-dimethylthiazol-2-yl)−2,5-diphenyl] tetrazolium bromide (MTT) for 1 h at 37 °C, lysed with DMSO and absorbance was determined at 490 nm (reference 690 nm).

### Statistical Analysis

**Fig. 1:**
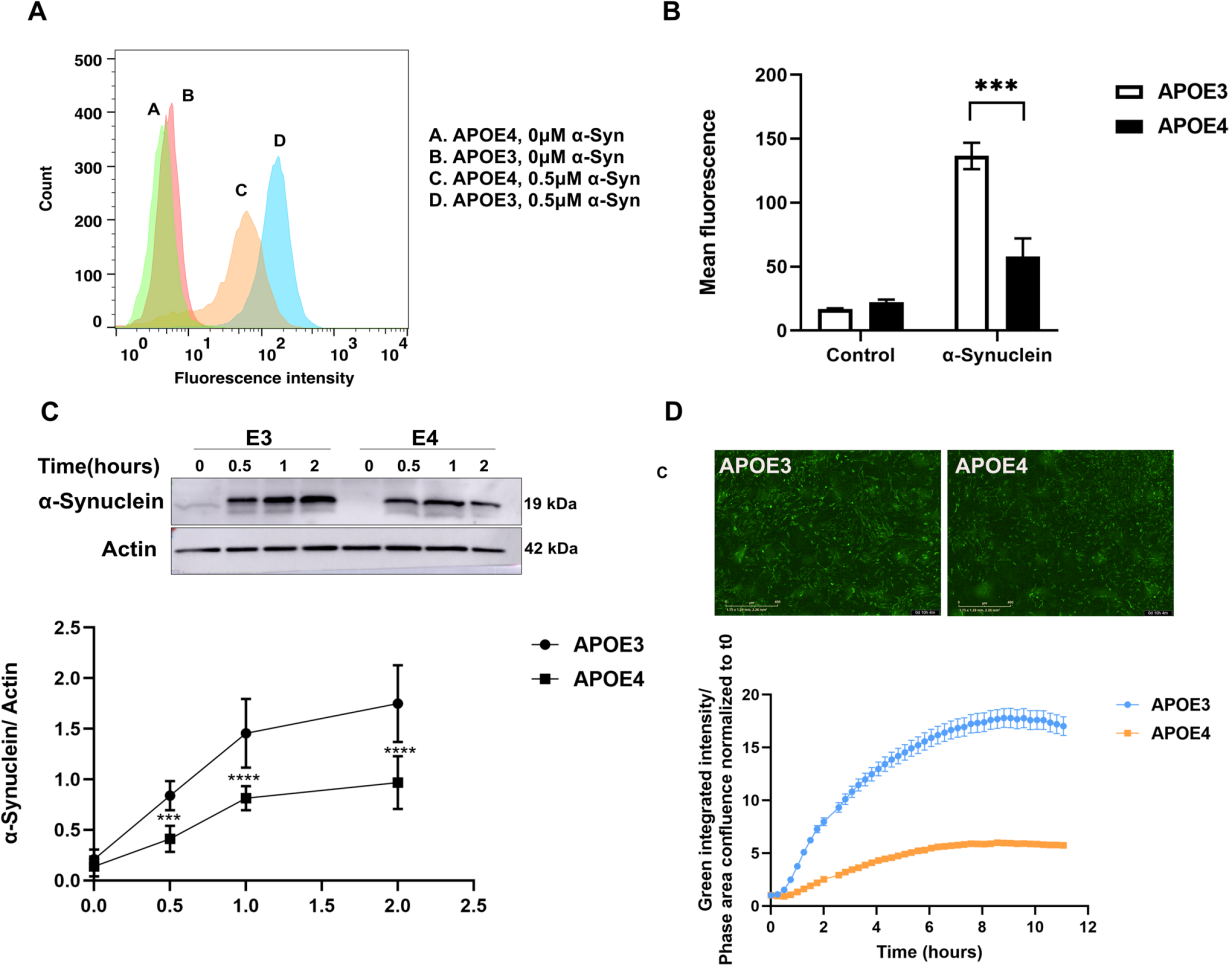
α-Synuclein uptake by *APOE3* compared to *APOE4*-expressing astrocytes. **A**, **B** *APOE3* and *APOE4*-expressing astrocytes were incubated with 0.5 μM α-synuclein labeled with FITC for 2 h. The amount of fluorescence inside the cells was quantified using flow cytometry. **A** Results are presented as the mean fluorescence of the treatment. **B** Results are presented as a bar plot of mean fluorescence; means ± SEM, ^***^p < 0.005, paired *t*-test (*n* = 5). **C** *APOE3* and *APOE4*-expressing astrocytes were incubated with 0.5 μM α-synuclein for 0–2 h. The levels of α-synuclein were measured by Western blot and normalized to actin. The lower panel depicts the densitometry of the results presented as fold induction of *APOE3* control; means ± SEM, ^***^q < 0.005, ^****^q < 0.001, repeated-measures two-way ANOVA with post hoc linear contrast and the two-stage FDR correction (*n* = 3). **D** *APOE3* and *APOE4*-expressing astrocytes were incubated with 0.5 μM α-synuclein labeled with FITC for 0–10 h, and intensity was measured and analyzed by live cell analysis (Incucyte®); extra sum-of-squares *F* test (*n* = 5)

All experiments were performed at least three times. Results are presented as mean ± SEM. Statistical significance was determined using Student’s *t*-test, one-sample *t*-test, multiple-paired *t*-tests followed by the false discovery rate (FDR) method, repeated-measures two-way ANOVA, and mixed-effect model followed by linear contrast and FDR correction, as indicated in the corresponding figure legend. To analyze the data in Figs. 1D and 4A, we first fit a centered third degree polynomial regression model to the datasets. We then used the extra sum-of-squares *F* test to test whether both datasets can be adequately explained using a single curve. Results were considered statistically significant when *p* < 0.05 or *q* < 0.05.

## Results

**Fig. 2:**
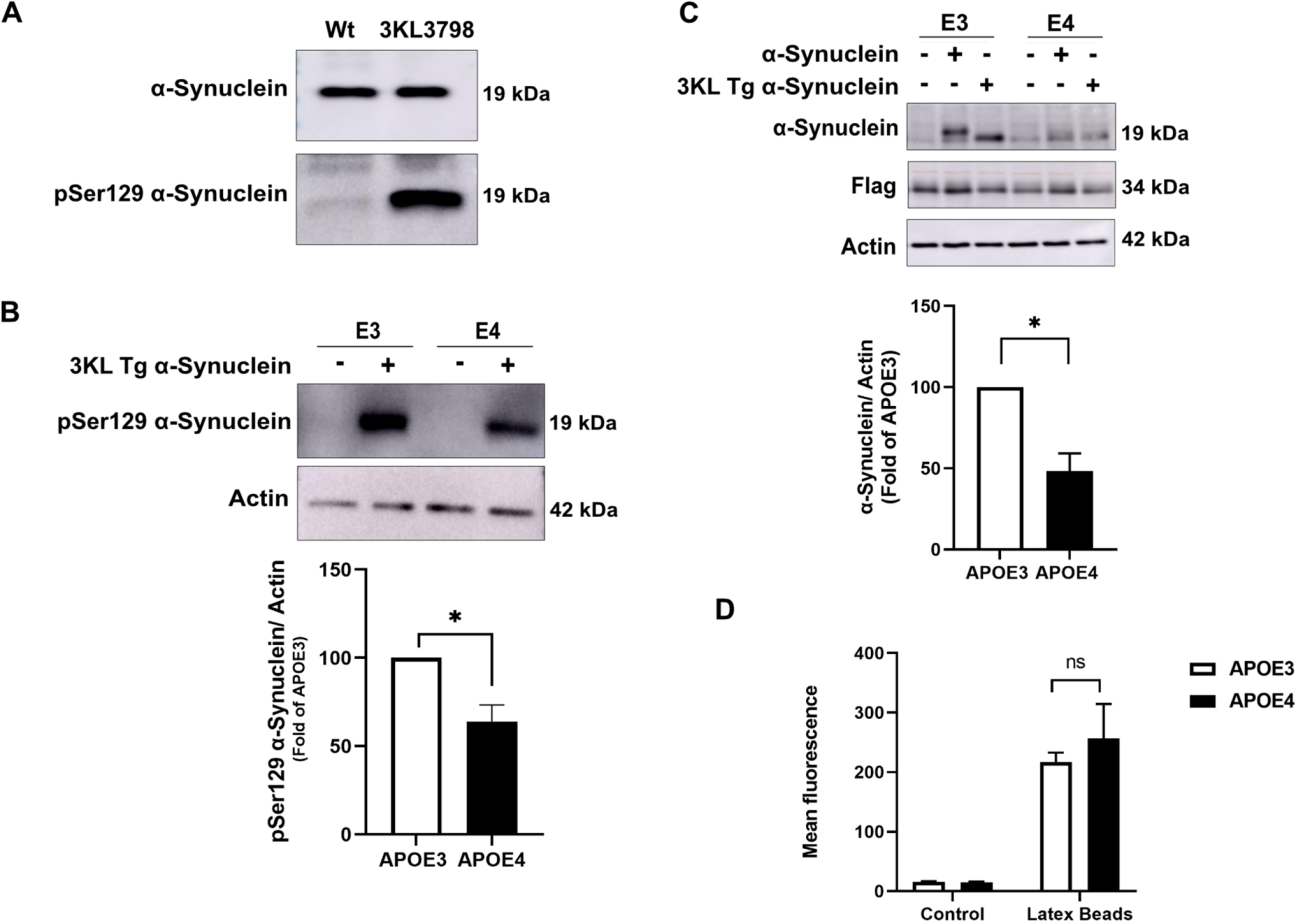
3KLTg α-synuclein uptake by *APOE3* compared to *APOE4*-expressing astrocytes. **A** Extracts from wild type compared to transgenic 3KL3798 mouse brain were prepared and the expression of WT and 3KLTg α-synuclein was detected by Western blot. **B** *APOE3* and *APOE4*-expressing astrocytes were treated with 1.5 μM extract containing pSer129 α-synuclein for 2 h. The levels of pSer129 α-synuclein were measured by Western blot and normalized to actin. The lower panel depicts the densitometry of the results which are presented as fold induction compared to *APOE3* control; means ± SEM, ^**^p < 0.01, one-sample *t*-test (*n* = 4). **C** U87 glioblastoma stably expressing Flag-*APOE3* and Flag-*APOE4* was incubated with 0.5 μM α-synuclein and 1.5 μM 3KLTg α-synuclein for 2 h. The levels of Flag*-APOE*, α-synuclein, and pSer129 α-synuclein were detected by Western blot. α-Synuclein and pSer129 α-synuclein levels were normalized to actin, and the lower panel depicts the densitometric analysis of the results presented as fold induction compared to *APOE3* control; means ± SEM, ^*^p < 0.05, ^**^p < 0. 01, one-sample *t*-test (*n* = 3). **D** *APOE3* and *APOE4*-expressing astrocytes were incubated with 1 μm diameter fluorescent beads for 2 h; means ± SEM, multiple-paired *t*-test with FDR correction (*n* = 3)

To examine whether *APOE4* expression in astrocytes affects glial characteristics involved in PD, *APOE3* or *APOE4*-expressing astrocytes were treated with fluorophore-labeled α-synuclein and the levels of α-synuclein uptake were measured by flow cytometry. As shown in Fig. 1A, B, the uptake of α-synuclein was significantly lower in *APOE4*- compared to *APOE3*-expressing astrocytes. To further study the extent of α-synuclein uptake by *APOE*-expressing cells, two additional methods were used. As shown in Fig. 1C, *APOE3* or *APOE4*-expressing astrocytes were treated with α-synuclein for the indicated time periods and then subjected to immunoblot analysis using anti-α-synuclein antibodies. As shown, the levels of α-synuclein were significantly lower in *APOE4*-expressing cells. We next examined the possibility that *APOE4* affects the uptake of soluble fluorescent α-synuclein using another method of live cell analysis (Incucyte®) during 12 h incubation. As shown in Fig. 1D, the uptake of α-synuclein by *APOE3* cells was significantly higher than the corresponding uptake by *APOE4* cells at various time points measured. Next, we set experiments to examine the ability of the cells to uptake mutant α-synuclein (3KLTg α-synuclein). The 3KL3798 mouse model expresses the human SNCA (synuclein alpha) with the E35K, E46K, and E61K mutations. 3KLTg α-synuclein brain cell lysates were prepared from 10-month-old 3KL3798 mice. The 3KL3798 mutations lead to a decrease in the tetramer-monomer ratio and to insoluble α-synuclein, which causes neurotoxicity. The insoluble α-synuclein is Ser129-phosphorylated (pSer129) and thus can be detected by immunoblotting [27]. As shown in Fig. 2A, the levels of endogenous α-synuclein were similar in lysates prepared from WT or transgenic animal brains. Nevertheless, the levels of pSer129 α-synuclein were detected only in lysates prepared from transgenic brains. Next, the uptake of mutant α-synuclein by *APOE3* and *APOE4*-expressing astrocytes was examined. The uptake of the mutant α-synuclein was significantly lower by *APOE4* cells (Fig. 2B). Figure 2C shows a comparison between the uptake of recombinant α-synuclein and mutant α-synuclein in U87 glioblastoma cells overexpressing human *APOE3* or *APOE4*. As shown, also in these cells the uptake by *APOE3*-expressing cells was significantly more efficient. Thus, consistent reductions were observed in recombinant and mutant α-synuclein across astrocytes and U87 cells (Figs. 1 and 2). Therefore, the results suggest that the uptake of α-synuclein is more efficient by *APOE3*- compared to *APOE4*-expressing astrocytes. As a control experiment, we determined the uptake of latex beads which showed similar uptake by both *APOE3* and *APOE4*-expressing astrocytes (Fig. 2D).

**Fig. 3:**
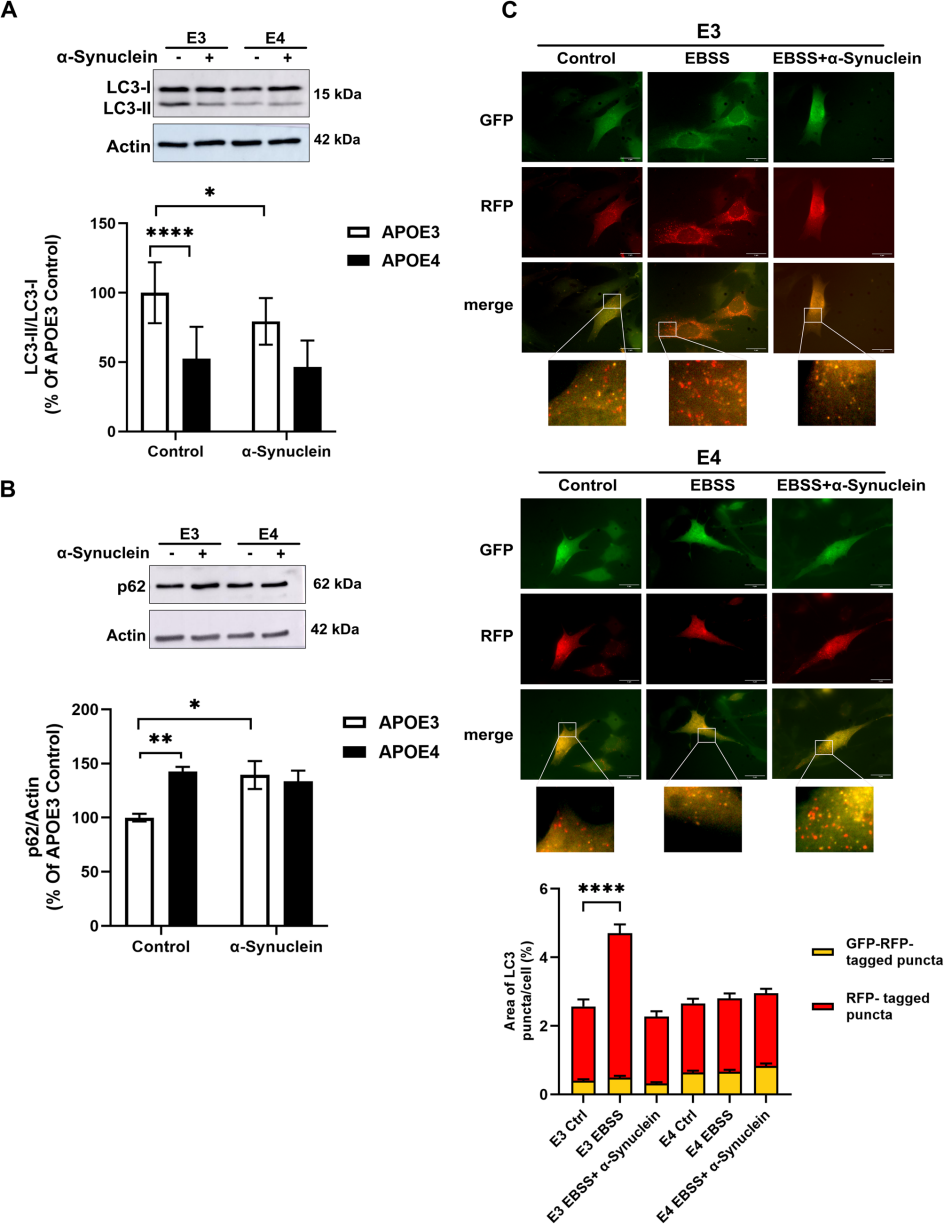
The effect of α-synuclein on autophagy in *APOE3* compared to *APOE4*-expressing astrocytes. **A**, **B** *APOE3* and *APOE4*-expressing astrocytes were incubated with 0.5 μM α-synuclein for 2 h. p62 and LC3 levels were detected by Western blot and normalized to actin. The lower panel depicts the densitometry of the results presented as fold induction compared to control; means ± SEM, ^*^ < 0.05, ^**^q < 0.005, ^****^q < 0.001, two-way ANOVA with post hoc linear contrast and the two-stage FDR correction (*n* = 6). **C** *APOE3* and *APOE4* astrocytes expressing LC3-EGFP-mRFP vector were treated with EBSS with or without 0.5 μM α-synuclein for 2 h. Upper panel, representative results (scale bars 25 μm); lower panel, ImageJ analysis of %area of LC3-EGFP-mRFP or LC3-mRFP-positive puncta; *n* > 50 cells/treatment; means ± SEM, ^****^q < 0.001, repeated-measures two-way ANOVA with post hoc linear contrast and the two-stage FDR correction

Since impaired autophagy was shown to be involved in PD pathology, specifically in the context of α-synuclein removal [28], we next measured autophagy levels in *APOE3*- and *APOE4*-expressing astrocytes in the presence or absence of α-synuclein. The protein Sequestosome 1, p62/SQSTM1 (p62) is involved in autophagic cargo recognition and degradation, including selection of α-synuclein [29, 30]. During autophagy, p62 levels are decreased and then synthesized to enable the progression of autophagy. As shown in Fig. 3A, the levels of p62 were significantly higher in *APOE4*-expressing cells, yet α-synuclein treatment increased the levels of p62 in the *APOE3* but not in *APOE4* cells in which the levels of p62 were high to begin with.

To further examine the effect of α-synuclein on autophagy, the levels of light chain 3 (LC3) following treatment were measured. As shown in Fig. 3B, α-synuclein treatment significantly reduced the levels of LC3-II in *APOE3*-expressing astrocytes. However, the levels of LC3-II were significantly lower in *APOE4* compared to *APOE3*-expressing astrocytes, and α-synuclein treatment had no effect on LC3-II levels in *APOE4* astrocytes. Furthermore, using the tandem LC3-GFP-RFP reporter, we found that following EBSS treatment, which enhances autophagy, a significant increase in autophagosome fusion with lysosomes was obtained in the *APOE3* astrocytes but not in the *APOE4* astrocytes (Fig. 3C). This effect in *APOE3* cells was inhibited by α-synuclein treatment.

**Fig. 4:**
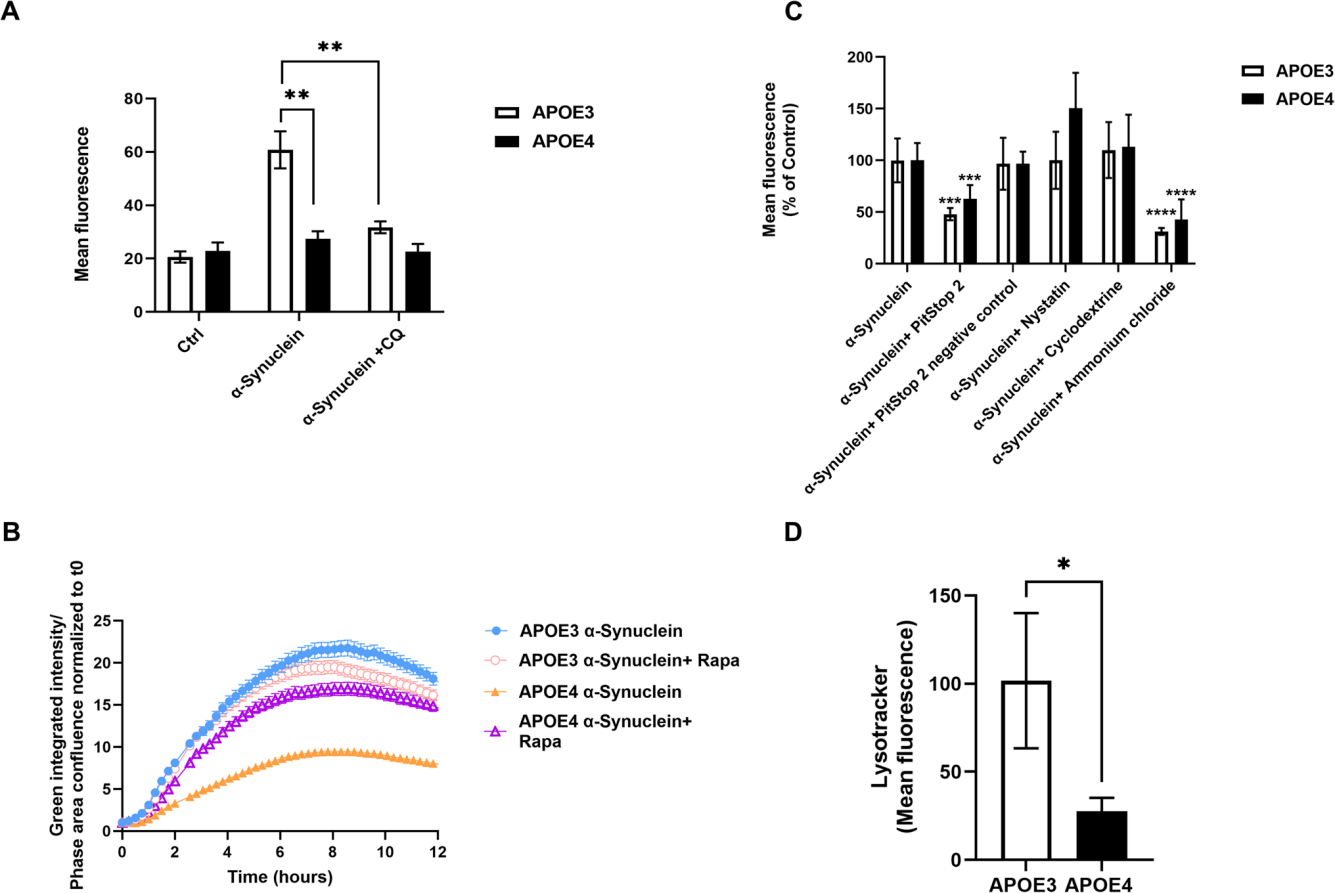
The effect of *apoe* alleles on α-synuclein-mediated uptake in response to autophagy and endocytosis manipulations. **A** *APOE3* and *APOE4*-expressing astrocytes were incubated with 0.5 μM α-synuclein labeled with FITC for 2 h in the presence or absence of 10 μM chloroquine. The amount of fluorescence inside the cells was quantified by flow cytometry; means ± SEM, ^**^q < 0.01, repeated-measures two-way ANOVA with post hoc linear contrast and the two-stage FDR correction (*n* = 3). **B** *APOE3* and *APOE4*-expressing astrocytes were incubated with 0.5 μM α-synuclein labeled with FITC for 0–12 h in the presence or absence of 100 nM rapamycin (Rapa) and fluorescence intensity in the cells was measured and analyzed by live cell analysis (Incucyte®), extra sum-of-squares *F* test (*n* = 3). APOE4 cells’ lysosomal pH is lower compared to APOE3 cells. **C** *APOE3* and *APOE4*-expressing astrocytes were incubated with 0.5 μM α-synuclein with or without 30 μM PitStop2, 25 μg/ml nystatin, 10 mM cyclodextrin, or 20 mM ammonium chloride for 30 min and the amount of fluorescence inside the cells was quantified using flow cytometry; means ± SEM, ^***^p < 0.005, ^****^p < 0.001, two-way mixed-effect ANOVA (*n* = 3). **D** *APOE3* and *APOE4*-expressing astrocytes were incubated with 60 nM LysoTracker for 30 min and the amount of fluorescence inside the cells was quantified using flow cytometry; means ± SEM, ^*^p < 0.05, ratio paired *t*-test (*n* = 3)

Next, we sought to examine whether autophagy inhibition would affect α-synuclein uptake. To this end, we used chloroquine, which decreases lysosomal acidity and thus inhibits autophagy. When cells were coincubated with chloroquine, the rate of α-synuclein uptake by *APOE3* cells was significantly reduced, but not by *APOE4* cells (Fig. 4A). To further establish the link between α-synuclein uptake and autophagic activity in the context of *APOE* expression, we treated *APOE3* or *APOE4* astrocytes with the autophagy inducer rapamycin and measured α-synuclein uptake. According to the results (Fig. 4B), rapamycin treatment increased the uptake of α-synuclein by the *APOE4* cells. Next, we examined whether inhibition of endocytic pathways affects α-synuclein uptake. As shown in Fig. 4C, Pitstop2, which inhibits clathrin-mediated endocytosis, significantly inhibited α-synuclein uptake in both *APOE3* and *APOE4*-expressing cells. Also, treatment with ammonium chloride, which increases lysosomal and endosomal pH, significantly inhibited α-synuclein uptake in both cell lines. Moreover, *APOE3*-expressing astrocytes showed higher lysosomal activity as indicated by higher lysotracker signal compared to *APOE4*-expressing cells (Fig. 4D). Thus, Pitstop2 and ammonium chloride inhibition of α-synuclein uptake in both cell types is consistent with endo-lysosomal pathway involvement.

**Fig. 5:**
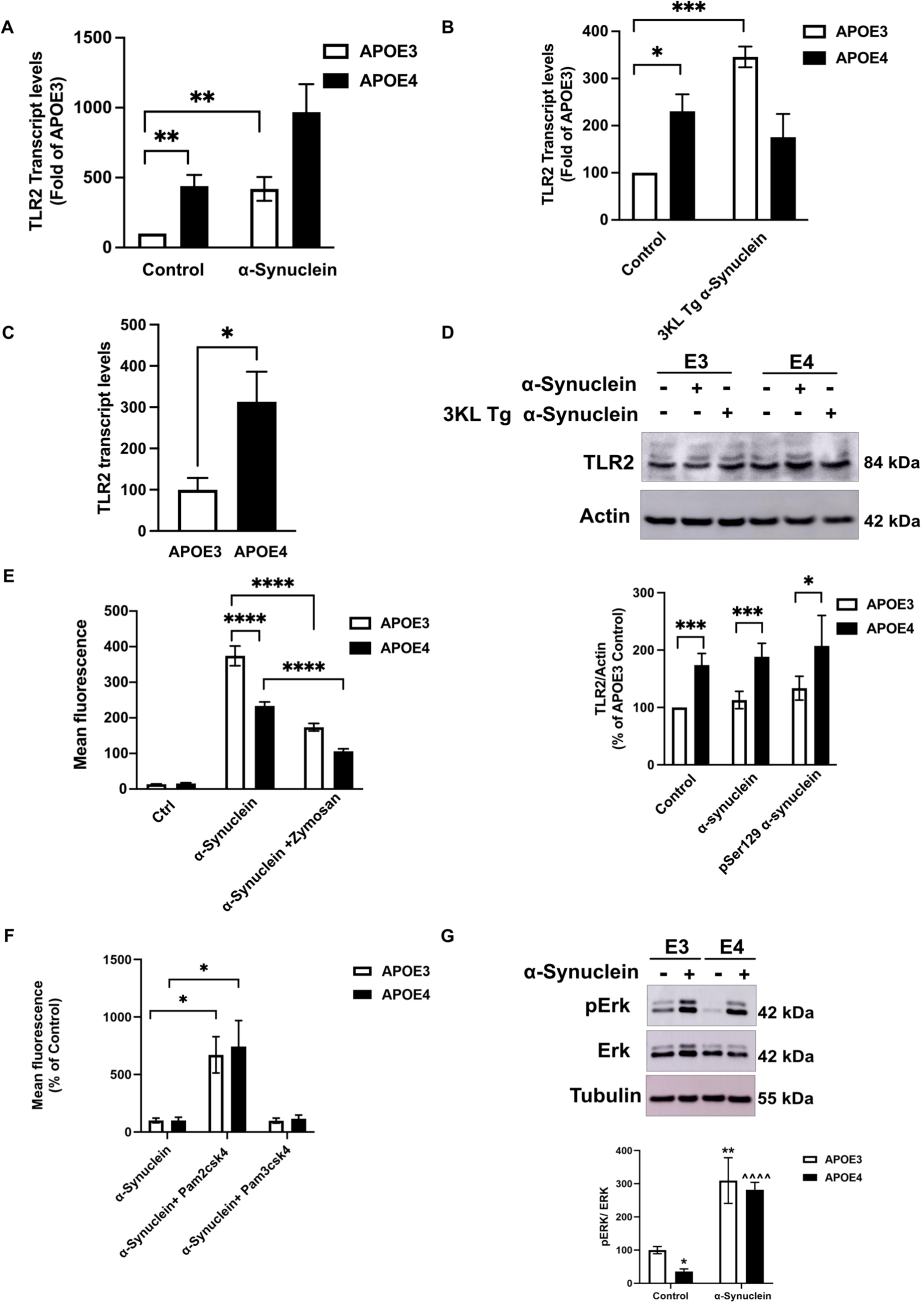
TLR2 expression in *APOE3* compared to *APOE4*-expressing astrocytes. **A**, **B** *APOE3* and *APOE4*-expressing astrocytes were treated with 0.5 μM α-synuclein or extract containing pSer129 α-synuclein for 2 h and subjected to real-time PCR of TLR2 expression (fold induction of *APOE3* control), means ± SEM, ^*^q < 0.05, ^**^q < 0.01, ^***^q < 0.005, multiple-paired *t*-test with FDR correction (*n* = 3). **C** 4–5-month-old target replacement APOE mouse (syn -/-) brain lysates were subjected to real-time PCR of TLR2 expression (fold of *APOE3*); means ± SEM, ^*^p < 0.05, one-tailed *t*-test (*n* = 3). **D** *APOE3* and *APOE4*-expressing astrocytes were treated with 0.5 μM α-synuclein or extract containing pSer129 α-synuclein for 2 h. The levels of TLR2 were measured by Western blot analysis and normalized to actin. The lower panel depicts the densitometry of the results presented as fold induction of *APOE3* control; means ± SEM, ^*^q < 0.05, ^***^q < 0.05, mixed-effect model and post hoc with linear contrast and the two-stage FDR correction. **E** *APOE3* and *APOE4*-expressing astrocytes were treated with 0.5 μM α-synuclein and 25 µg/ml Zymosan for 2 h. The amount of fluorescence inside the cells was quantified by flow cytometry; means ± SEM, ^****^q < 0.001, two-way ANOVA with post hoc linear contrast and the two-stage FDR correction (*n* = 3). **F** *APOE3* and *APOE4*-expressing astrocytes were incubated with 0.5 μM α-synuclein and with 10 μM Pam2Csk4 or Pam3Csk4 for 30 min, and the amount of fluorescence inside the cells was quantified using flow cytometry; means ± SEM, ^*^p < 0.05, two-way ANOVA (*n* = 3). **G** *APOE3* and *APOE4*-expressing astrocytes were treated with 0.5 μM α-synuclein for 2 h. The levels of pErk and Erk were measured by Western blot analysis and normalized to Tubulin. The lower panel depicts the densitometry of the results presented as fold induction of *APOE3* control; means ± SEM, ^*^p < 0.05, ^**^p < 0.01 compared to *APOE3* control, ^^^^^^*p* < 0.001 compared to *APOE4* control, two-way ANOVA (*n* = 3)

Toll-like receptor 2 (TLR2) has been previously implicated in α-synuclein uptake [31, 32]. Therefore, we next examined TLR2 mRNA levels following α-synuclein and 3KLTg α-synuclein treatments in *APOE3* and *APOE4*-expressing astrocytes. As shown in Fig. 5A, B, the basal TLR2 mRNA levels were higher in *APOE4*-expressing astrocytes, while both α-synuclein and 3KLTg α-synuclein treatments increased TLR2 levels in *APOE3* cells. We also compared the TLR2 mRNA levels in mRNA prepared from brains of *APOE3* and *APOE4* target replacement mice [26]. As shown in Fig. 5C, the TLR2 mRNA levels in *APOE4* were significantly higher than in *APOE3*. Next, we sought to examine the protein levels of TLR2 in *APOE3* and *APOE4*-expressing astrocytes with or without α-synuclein or 3KLTg α-synuclein treatments. TLR2 levels were significantly higher in *APOE4*-expressing astrocytes compared to *APOE3*. However, no significant changes in TLR2 protein levels were observed following α-synuclein or 3KLTg α-synuclein treatments. Next, we examined the effect of TLR activators on α-synuclein uptake. Pam2CSK4 which activates specifically TLR2/TLR6 significantly increased α-synuclein uptake by both *APOE3* and *APOE4*-expressing cells (Fig. 5F). Pam3CSK4, which is the activator of TLR1/TLR2, had no effect on *APOE3* and *APOE4*-expressing astrocytes. Zymosan, however, which binds TLR2 and TLR6, significantly inhibited α-synuclein uptake in both *APOE3* and *APOE4*-expressing astrocytes. One explanation for these results can rely on the fact that Zymosan complex is engaged to TLR2 and non-TLR receptors (such as Dectin-1) whereas the selective TLR2/6 activation by Pam2CSK4 boosts astrocytic endocytic machinery and increases α-synuclein internalization. Next, we examined the levels of Erk phosphorylation (pErk), a downstream effector of TLR2. As shown in Fig. 5G, at the basal level pErk was lower in *APOE4* cells compared to *APOE3* cells. Following α-synuclein incubation, the levels of pErk were significantly increased in both cell types.

**Fig. 6:**
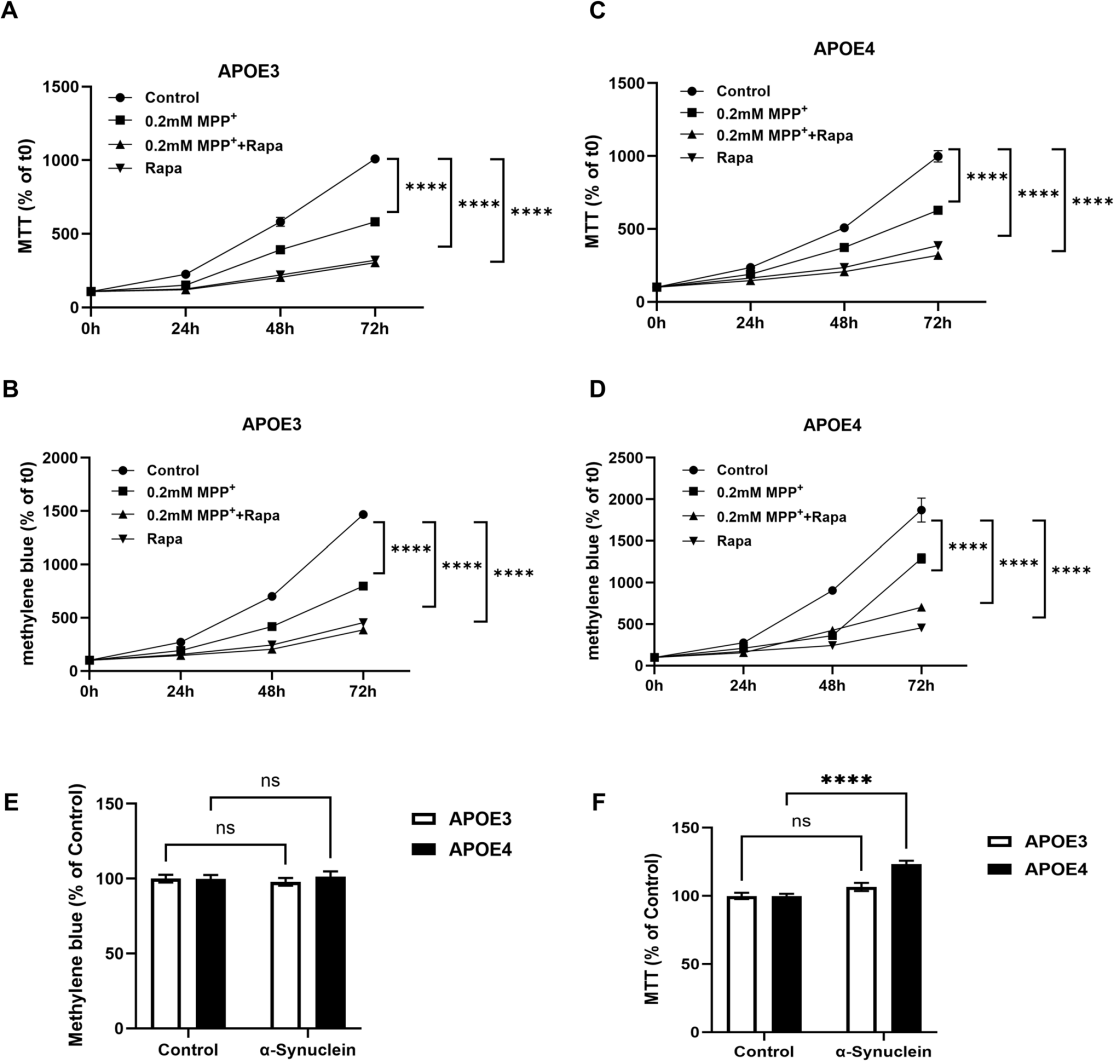
The effect of MPP^+^ on cell viability. *APOE3* and *APOE4*-expressing astrocytes were treated with 0.2 mM MPP^+^ in the presence or absence of 100 nM rapamycin for 24–72 h and cell viability was determined using MTT (**A** and **C**) and methylene blue (**B** and **D**) assays; means ± SEM, ^****^p < 0.001, two-way ANOVA (*n* = 3). **E**, **F** *APOE3* and *APOE4*-expressing astrocytes were treated with 0.5 μM α-synuclein for 24 h, and cell viability was determined using methylene blue and MTT assays, means ± SEM, ^****^p < 0.001, two-way ANOVA with mixed effects (*n* = 3)

Having shown that α-synuclein uptake and α-synuclein effects on autophagy differ between *APOE3* and *APOE4-*expressing cells, we next set experiments to examine the effect of α-synuclein and MPP^+^, a known neurotoxin used in models of PD, on cell number and mitochondrial activity in cells expressing the *APOE* isoforms. As shown in Fig. 6A–D, MPP^+^ treatment had an effect on cell viability. Under the conditions tested here, rapamycin reduced astrocyte cell number in *APOE3* and *APOE4* cells as well as in a combined treatment with MPP^+^ (Fig. 6A–D). Next, we examined the effect of the α-synuclein treatment on cell viability. As shown in Fig. 6E, F, α-synuclein treatment had no effect on cell viability of both *APOE3* and *APOE4-*expressing astrocytes as judged by methylene blue assay. However, α-synuclein treatment significantly increased mitochondrial activity in *APOE4* cells as judged by the MTT assay.

**Fig. 7:**
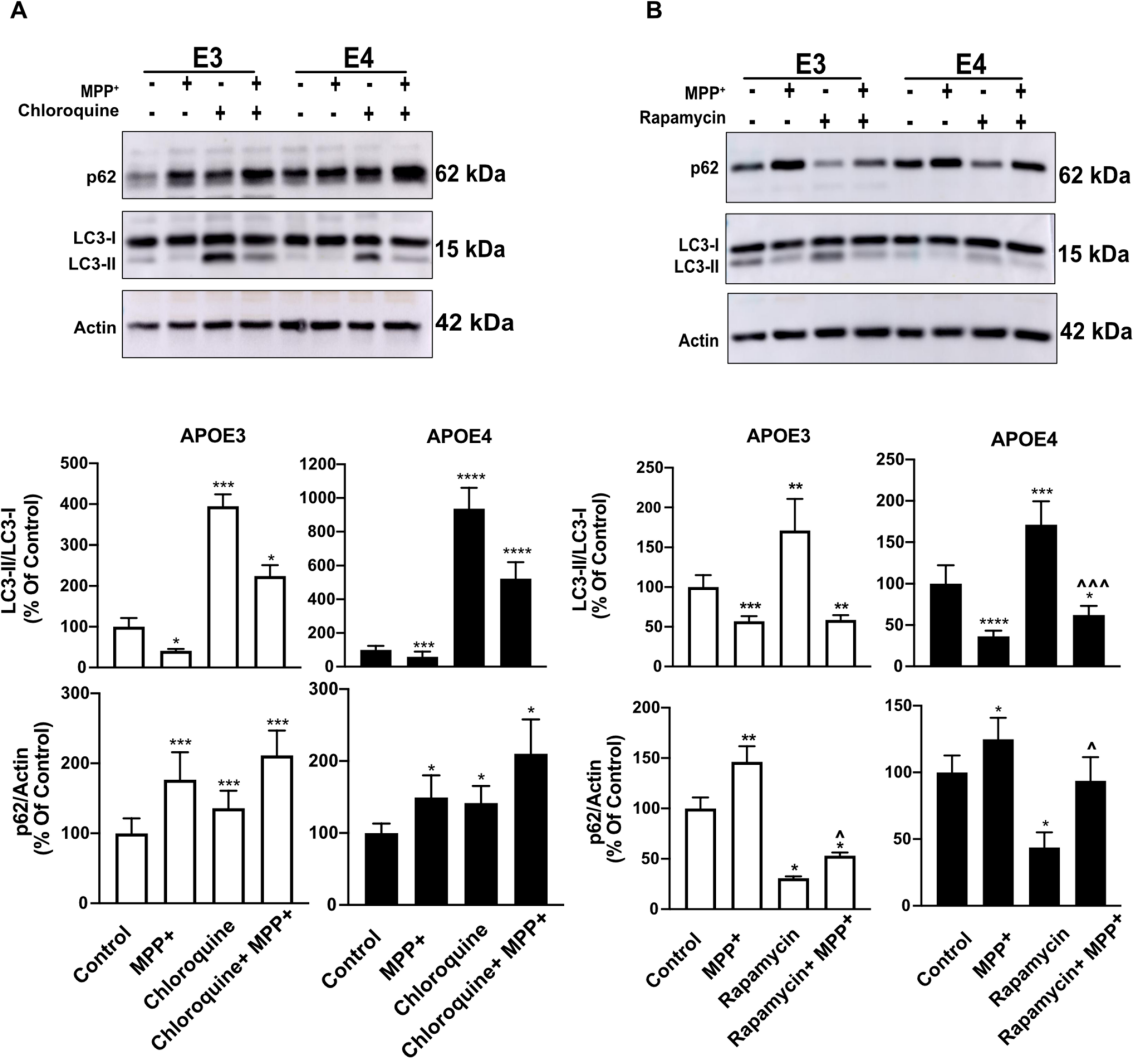
The effect of MPP^+^, rapamycin, and chloroquine on autophagy-related protein levels. *APOE3* and *APOE4*-expressing astrocytes were treated with 0.2 mM MPP^+^ in the presence or absence of **A** 10 μM chloroquine for 24 h and **B** 100 nM rapamycin for 24 h. p62 and LC3 levels were detected by Western blot and normalized to actin. The lower panel depicts the densitometry of the results presented as fold induction related to control; means ± SEM, ^*^q < 0.05, ^***^q < 0.005, ^****^q < 0.001, compared to control, ^^^*q* < 0.05, MPP^+^ compared to rapamycin + MPP^+^. Repeated-measures two-way ANOVA with post hoc linear contrast and the two-stage FDR correction (*n* = 5)

Next, we examined the effect of MPP^+^ treatment on autophagy using autophagy modulators (Fig. 7). To assess the effect of autophagy inhibition, we examined the effect of the MPP^+^ treatment with and without chloroquine. As demonstrated in Fig. 7A, MPP^+^ treatment significantly reduced the levels of LC3-II and increased the levels of p62 in *APOE3* and *APOE4*-expressing cells. Inhibition of autophagy by chloroquine treatment increased LC3-II levels in both *APOE3* and *APOE4*-expressing astrocytes (Fig. 7A). However, MPP^+^ significantly inhibited the effect of chloroquine on *APOE3* and *APOE4*-expressing astrocytes as judged by LC3-II levels. In addition, p62 levels were significantly increased after treatments with either MPP^+^, chloroquine, or their combination.

In addition, we examined the effect of autophagy induction by rapamycin on MPP^+^-induced changes in p62 and LC3-II levels. As shown in Fig. 7B, p62 levels increased following MPP^+^ treatment and decreased following rapamycin treatment. Combined treatment of MPP^+^ and rapamycin reduced p62 levels compared to MPP^+^ treatment alone in both *APOE3* and *APOE4-*expressing astrocytes, with more pronounced effect on *APOE3-*expressing cells. The LC3-II/LC3-I ratio was significantly higher in *APOE3* compared to *APOE4-*expressing astrocytes. LC3-II/LC3-I ratio was reduced by MPP^+^ treatment and was higher following rapamycin treatment. The ratio was increased in *APOE4* cells in the combined treatment compared to MPP^+^ treatment alone.

## Discussion

Parkinson’s disease (PD) is a progressive neurodegenerative disease characterized by tremor, rigidity, and impaired movement, speech, writing, and body posture. It is also characterized by the presence of Lewy bodies, containing α-synuclein aggregates at the substantia nigra, leading to degeneration of dopaminergic neurons [1]. Here we examined the possibility that *apoe4* allele which is a risk factor for Alzheimer’s disease (AD) is also involved in α-synuclein-mediated effects, thus promoting PD pathogenesis. Our findings suggest that following exposure to α-synuclein, *APOE4*-expressing astrocytes demonstrate decreased uptake of α-synuclein compared to *APOE3* astrocytes. Also, α-synuclein treatment inhibited autophagy in *APOE3* but not in *APOE4* cells which their basal autophagy was reduced to begin with. Treatment with autophagy inhibitor chloroquine decreased α-synuclein uptake in *APOE3* astrocytes, and autophagy enhancer rapamycin increased the uptake of α-synuclein by *APOE4* astrocytes. These findings might indicate that altered autophagy is involved in the effects of *APOE4* in PD. In addition, *APOE4* cells express higher TLR2 levels compared to *APOE3* cells. Moreover, MPP^+^ treatment inhibited autophagy which was partially rescued by rapamycin treatment. MPP^+^ treatment also reduced cell viability in both *APOE3* and *APOE4* cells.

Autophagy is a lysosome-mediated degradation process that eliminates damaged cellular components, including damaged organelles and protein aggregates [33]. Studies suggest that autophagy plays a key role in PD pathogenesis [34]. It was also recently shown that autophagy is impaired in astrocytes expressing *APOE4* compared to *APOE3*. Under several autophagy-inducing conditions, astrocytes expressing *APOE4* exhibited lower autophagic flux compared to astrocytes expressing *APOE3* [13]. Similarly, we have shown that *APOE4*-expressing astrocytes exhibit higher p62 levels and reduced LC3-II levels, indicating impaired autophagy. However, α-synuclein treatment increased p62 levels and reduced LC3-II levels in *APOE3*-expressing astrocytes but not in *APOE4*. It might be that *APOE4* cells which have impaired autophagy to begin with are less affected by α-synuclein treatment compared to *APOE3*-expressing cells.

It was previously demonstrated that *APOE* genotype directly affects α-synuclein pathology in A53T α-synuclein transgenic mice (A53T) expressing the various *apoe* alleles [20]. We have shown previously that the uptake of amyloid-beta is impaired in *APOE4* astrocytes, and these cells also eliminate plaques less effectively than the corresponding *APOE3* [13]. Here, we compared the ability of *APOE3* and *APOE4*-expressing astrocytes to uptake α-synuclein. Our results show that *APOE4*-expressing astrocytes exhibit less effective uptake of α-synuclein and mutant α-synuclein compared to *APOE3* astrocytes. Next, we wanted to examine the possibility that autophagy is involved in the apparent differences in α-synuclein uptake. We found that under autophagy inhibiting conditions, α-synuclein uptake by *APOE3* astrocytes was decreased and the induction of autophagy with rapamycin enhanced α-synuclein uptake by *APOE4* astrocytes. Using the levels of LC3 proteins as a marker for autophagy activation and the levels of p62 as a marker of autophagy-mediated protein degradation, we showed that astrocytes expressing *APOE3 *(but not *APOE4*) exhibit lower autophagy following α-synuclein treatment, as demonstrated by the increased levels of p62 and decreased levels of LC3-II/LC3-I. In *APOE4* astrocytes, the autophagy was lower than in *APOE3* to begin with. These results are consistent with previous findings demonstrating that α-synuclein inhibits autophagy [35]. In addition, we demonstrated that inhibition of autophagy, by chloroquine, decreases the uptake of α-synuclein by *APOE3*-expressing astrocytes. Thus, these results may suggest that inhibition of autophagy may affect the uptake of α-synuclein. Also, we can assume that the effect of autophagy inhibition on *APOE3*-expressing astrocytes was more pronounced because autophagy was impaired in APOE4-expressing cells. Moreover, increase in the α-synuclein uptake levels in *APOE4* astrocytes following rapamycin treatment suggests that enhancement of autophagy can partially correct the defects of autophagy in *APOE4* cells.

Since α-synuclein uptake and autophagic activity were different in *APOE3* and *APOE4*-expressing cells, we examined the involvement of the endosomal-lysosomal system. Interestingly, we found that *APOE4* cells exhibited impaired lysosomal acidity, which may affect α-synuclein degradation and uptake. We also found that clathrin-mediated endocytosis is involved in α-synuclein uptake in both cell lines. Given that TLR2 has been shown to recognize various ligands, including lipoproteins, and was previously suggested to be involved in α-synuclein uptake [31, 32], we next examined TLR2 expression in *APOE3* and *APOE4*-expressing astrocytes. We found that the mRNA as well as the protein levels of TLR2 were significantly higher in *APOE4* compared to *APOE3*-expressing astrocytes. Nevertheless, α-synuclein and 3KLTg α-synuclein treatments increased only the TLR2 mRNA level in *APOE3* astrocytes. No significant change in TLR2 protein levels was found following α-synuclein and 3KLTg α-synuclein treatments. These results are in agreement with previous studies showing that TLR2 mRNA and protein levels are significantly upregulated in the brains of PD patients and in animal models of synucleinopathy [31, 32]. Also, it was shown that TLR2 activation impairs the autophagy-lysosomal pathway in astrocytes and affects neurodegeneration and α-synuclein pathology [36]. Zymosan, which engages broader phagocytic pathways (including Dectin-1 and complement receptors) beyond TLR2, significantly inhibited α-synuclein uptake in both *APOE3* and *APOE4*-expressing astrocytes. However, the TLR2 agonist Pam2CSK4 increased α-synuclein uptake in both cell types as was previously demonstrated in brain resident cell including astrocytes [32]. Since *APOE4* cells express high TLR2 receptor levels, they may be more sensitive to α-synuclein leading to an increased level of pErk. However, these cells may be functionally impaired in clearing α-synuclein because the autophagic/lysosomal machinery is compromised.

Taken together, the results presented suggest that the pathological effects of *APOE4* in astrocytes may be mediated by impaired autophagy and by the concomitant impaired ability of the cells to remove α-synuclein. Whether *APOE4*-expressing astrocytes exacerbate neuronal damage remains unknown and needs further study. Nevertheless, our observations raise the possibility that autophagy-targeting strategies could improve astrocyte clearance of α-synuclein, thus may provide a novel approach for treatment of *APOE4*-related brain pathology in PD.

## Supplementary Information

Below is the link to the electronic supplementary material.

ESM 1 (DOCX 873 KB)

## Data Availability

No datasets were generated or analysed during the current study.
