# Correction: APOE4-Expressing Astrocytes Exhibit Parkinson’s Disease–Related Pathology

**DOI:** 10.1007/s12035-026-06067-5

**Published:** 2026-07-14

**Authors:** Lior Nechushtai, Anna Popov, Roni Haklai, Daniel M. Michaelson, Dan Frenkel, Ronit Pinkas-Kramarski

**Affiliations:** School of Neurobiology, Biochemistry and Biophysics, Department of Neurobiology, Tel-Aviv University, Ramat-Aviv, 69978 Israel

**Correction: Molecular Neurobiology (2026) 63:690**

10.1007/s12035-026-05996-5

In the original version of this article, the given and family names of authors were incorrectly structured as N. Lior, P. Anna, H. Roni, M. M. Daniel, F. Dan and P. K. Ronit. The correct citation should be: L Nechushtai, A Popov, R Haklai, DM Michaelson, D Frenkel, R Pinkas-Kramarski.

The original article has been corrected.

