# Supplementary material for: APOE4-Expressing Astrocytes Exhibit Parkinson’s Disease–Related Pathology
Source: Mol Neurobiol. 2026 Jun 11;63(1):690. doi: 10.1007/s12035-026-05996-5 (PMC13260029; doi:10.1007/s12035-026-05996-5)
Supplement: Supplementary file 1 — (DOCX 873 KB) [file 12035_2026_5996_MOESM1_ESM.pdf]

Supplementary file

Figure 1 C:

Upper part of the blot:

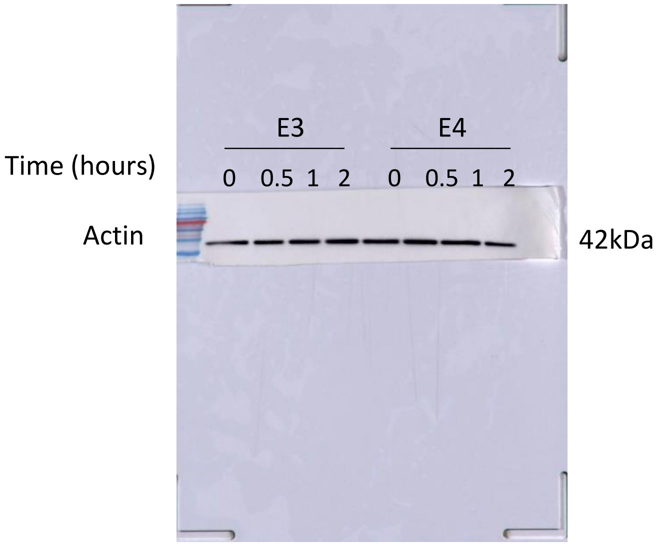

Lower part of the blot:

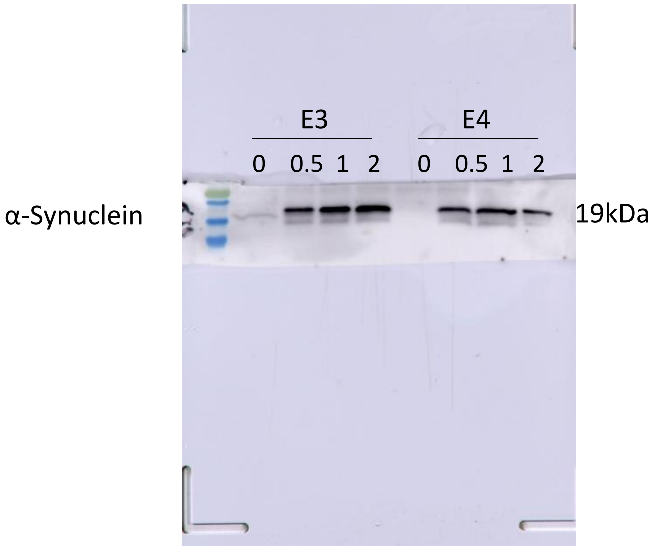

Figure 2A:

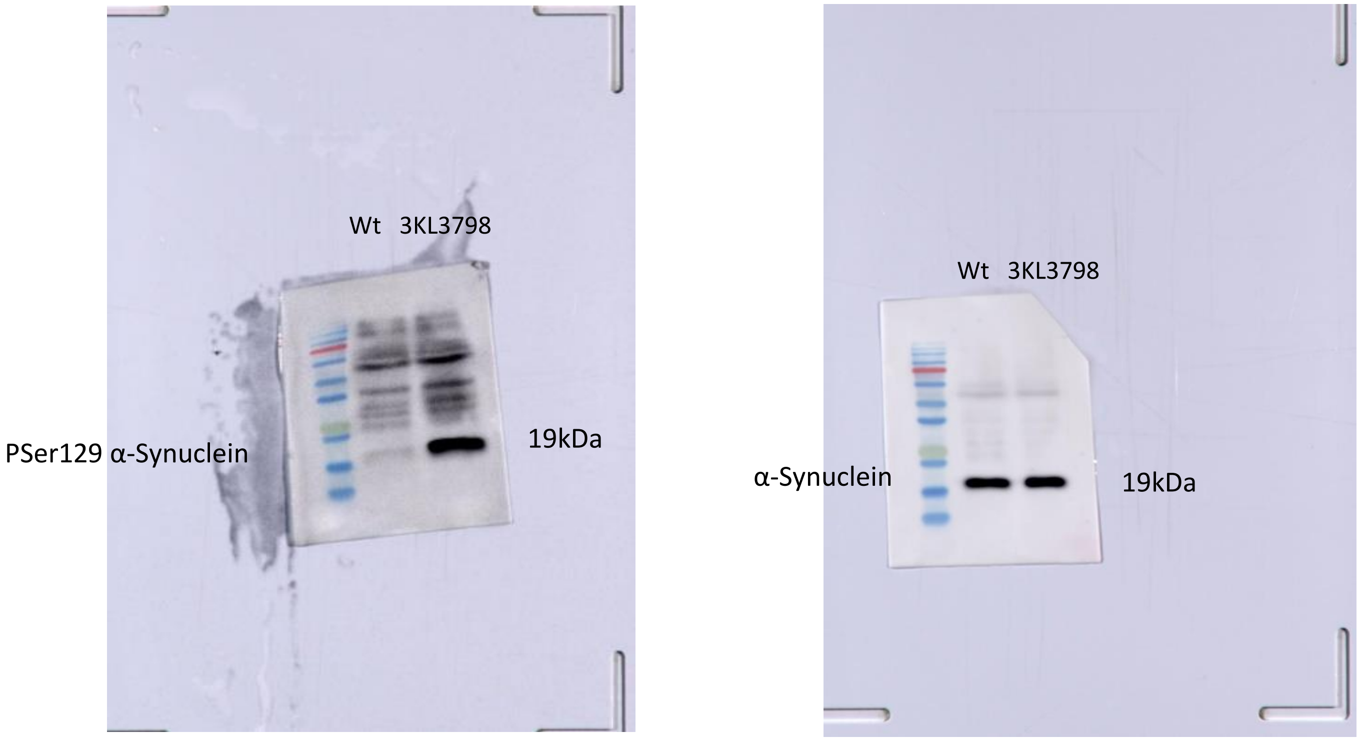

Figure 2B:

The original gel: after stripping :

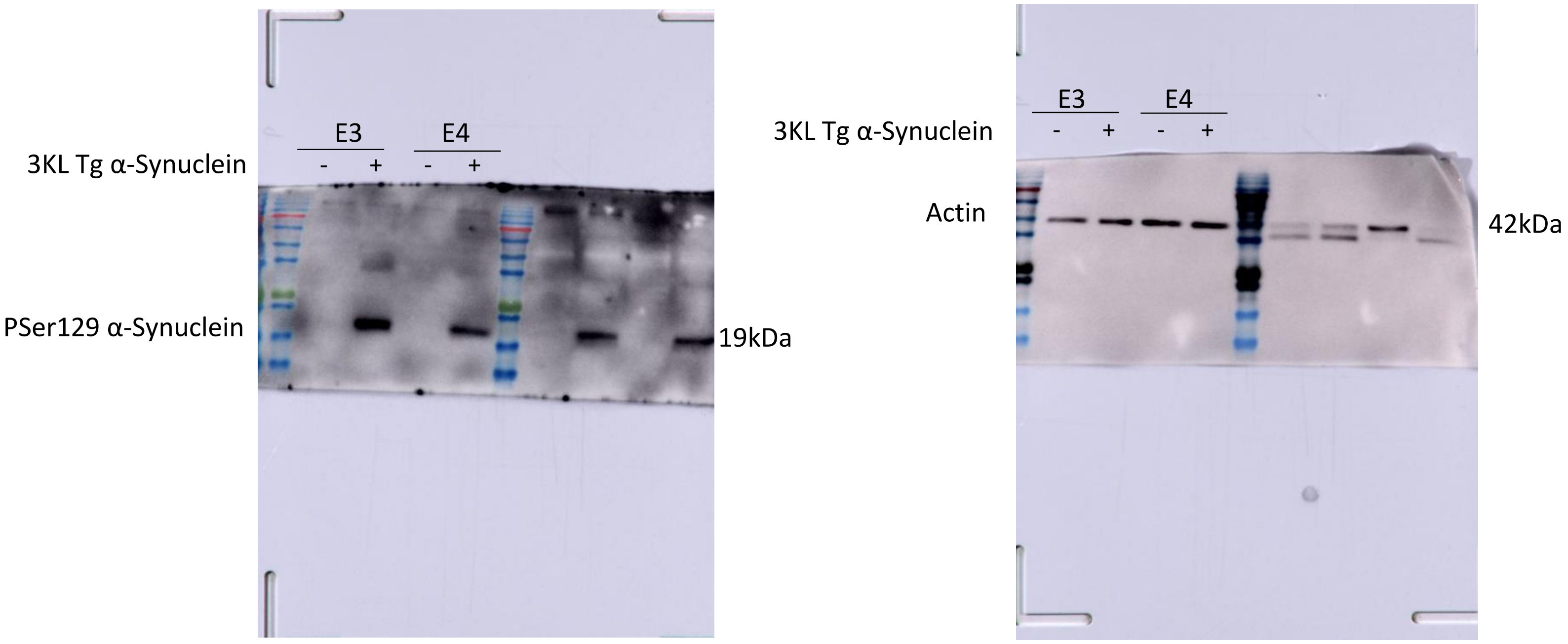

Figure 2C:

Lower gel:

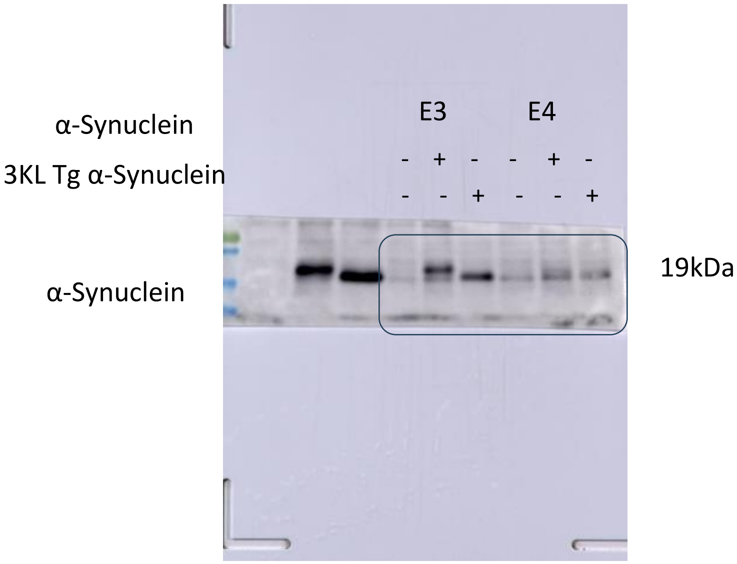

Upper gel:

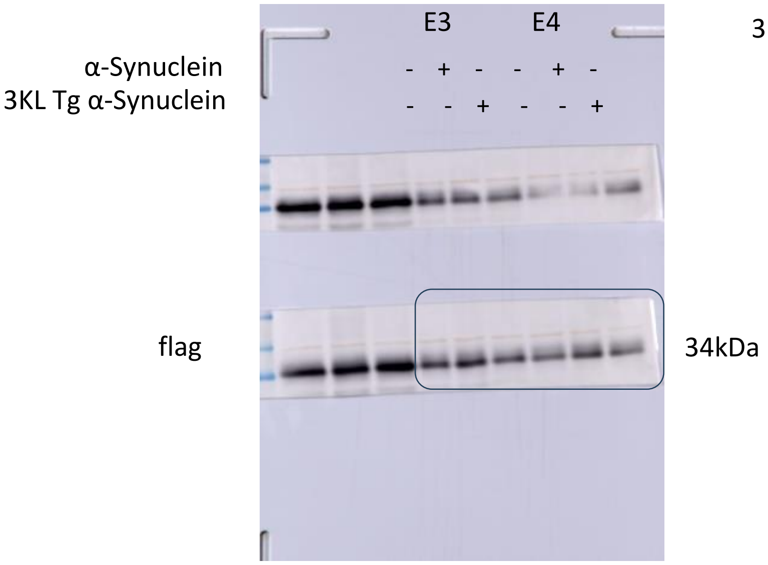

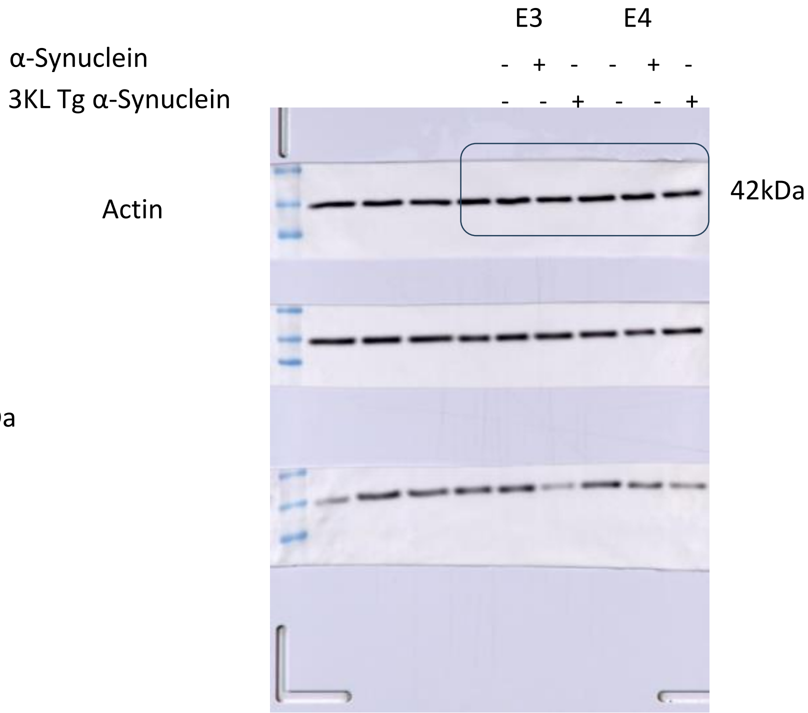

Upper gel (following membrane stripping):

Figure 3A:

LC3-I
LC3-II

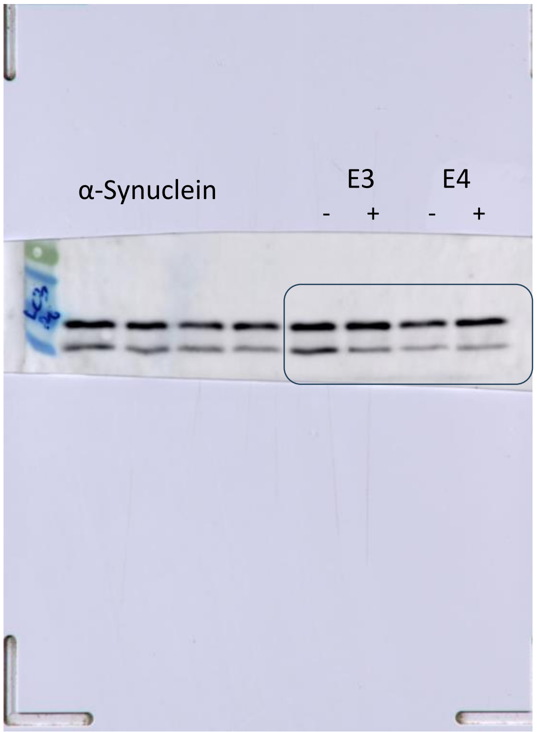

15 kDa

Actin

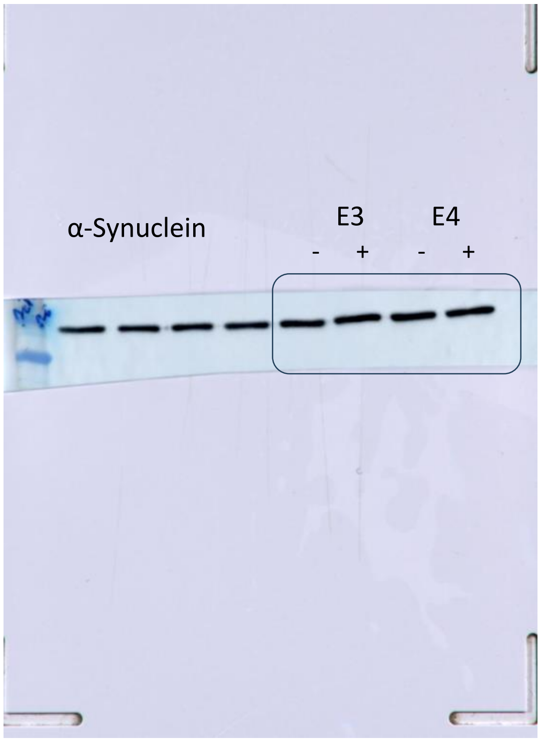

42kDa

Figure 3B:

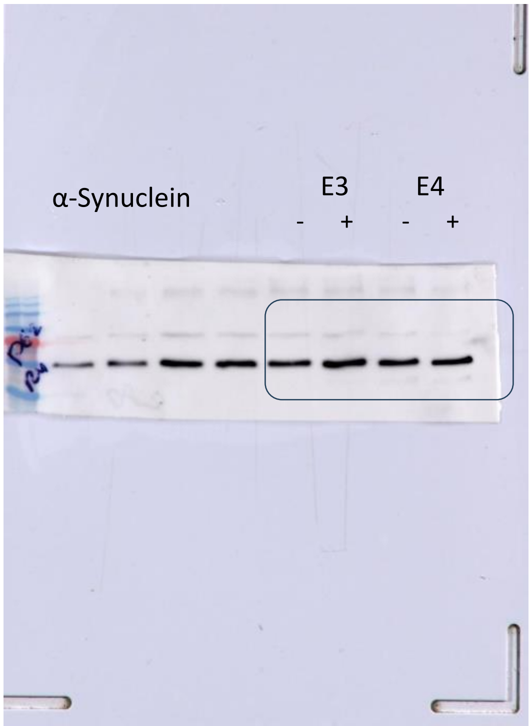

p62

62kDa

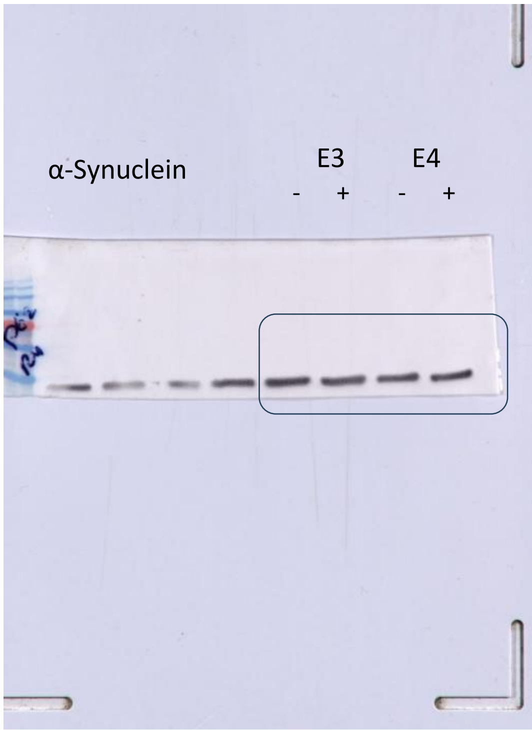

Actin

42kDa

Figure 5D:

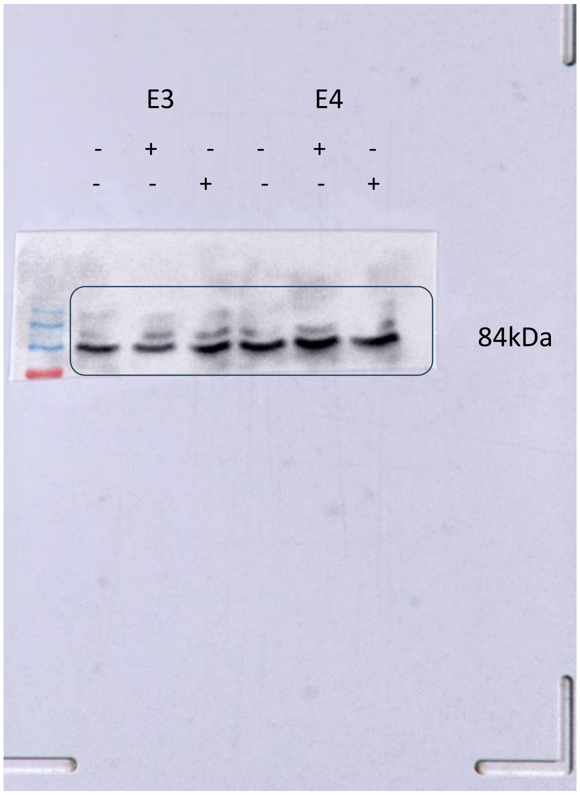

E3
E4
α-Synuclein
- + - - + -
3KL Tg α-Synuclein
- - + - - +
TLR2
84kDa

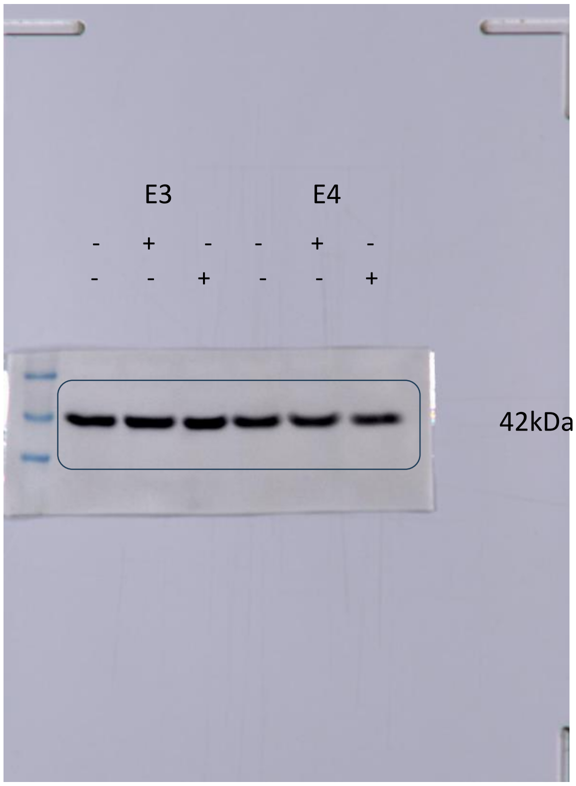

E3
E4
α-Synuclein
- + - - + -
3KL Tg α-Synuclein
- - + - - +
Actin
42kDa

Figure 5G:

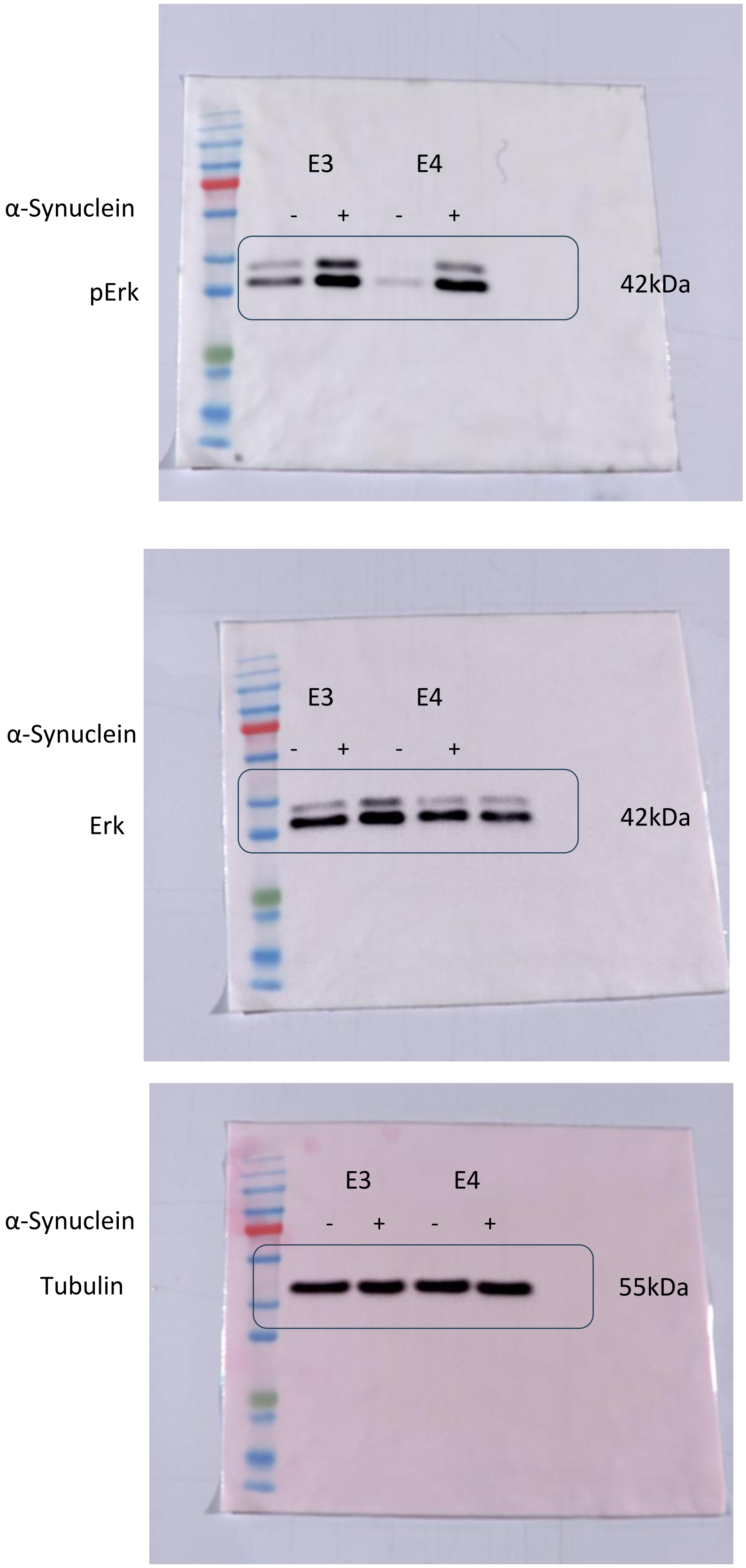
α-Synuclein
pErk
E3
E4
- + - +
42kDa
α-Synuclein
Erk
E3
E4
- + - +
42kDa
α-Synuclein
Tubulin
E3
E4
- + - +
55kDa

Figure 7A:

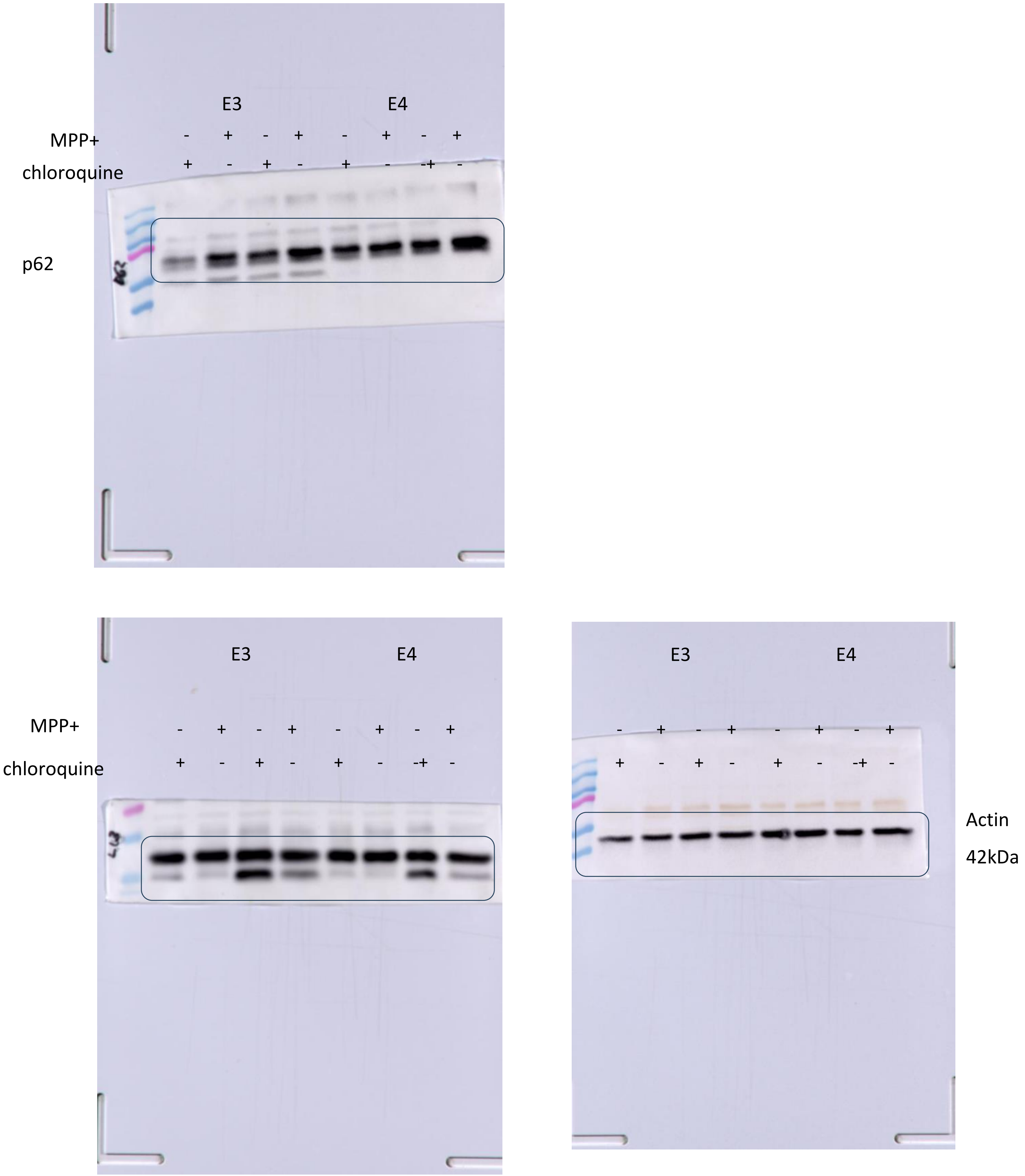
E3
E4
MPP+
- + - + - + - +
chloroquine
+ - + - + - -+ -
p62
E3
E4
MPP+
- + - + - + - +
chloroquine
+ - + - + - -+ -
E3
E4
- + - + - + - +
+ - + - + - -+ -
Actin
42kDa

Figure 7B:

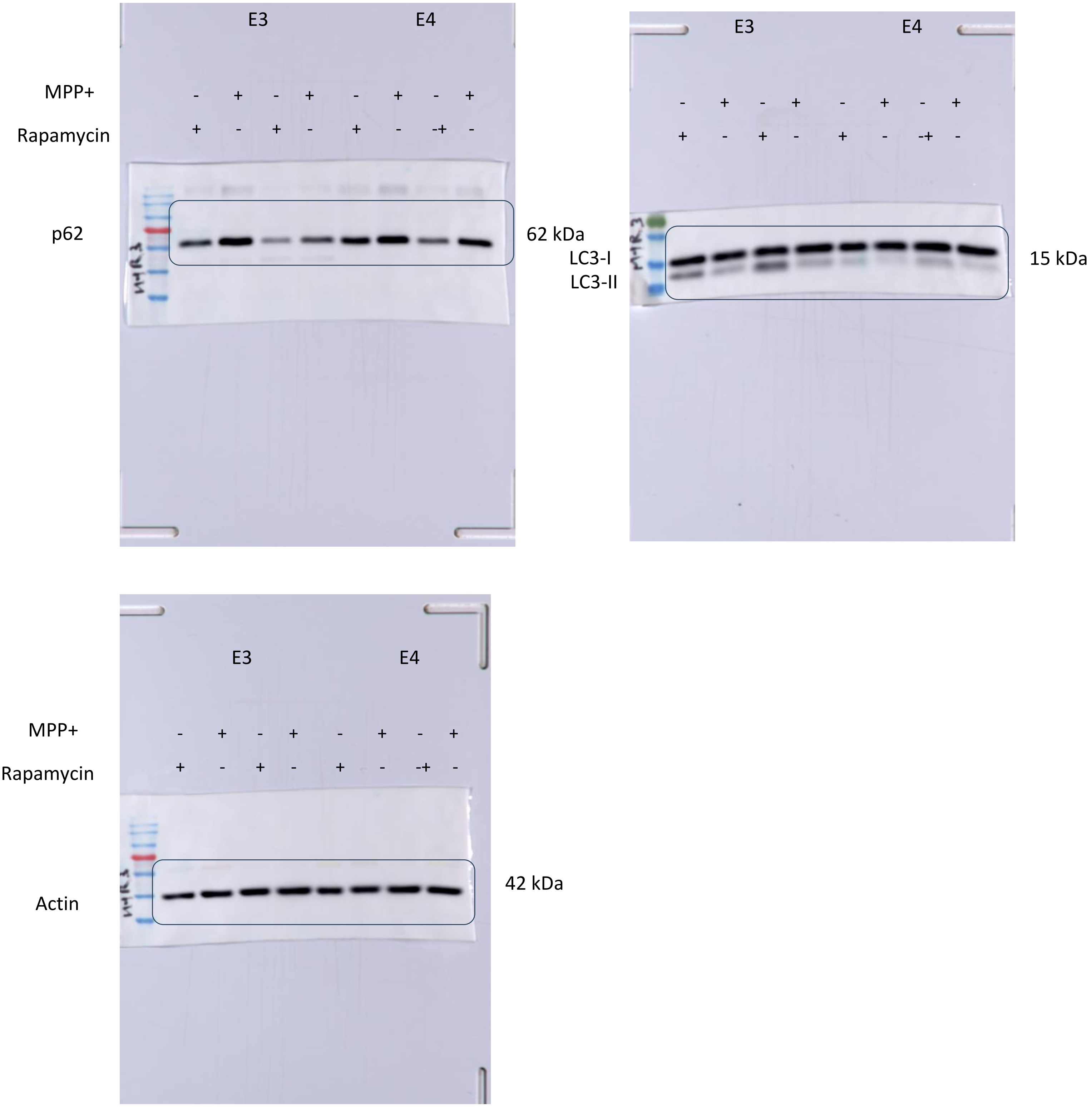
E3
E4
MPP+
- + - + - + - +
Rapamycin
+ - + - + - -+ -
p62
62 kDa
E3
E4
- + - + - + - +
+ - + - + - -+ -
LC3-I
LC3-II
15 kDa
E3
E4
MPP+
- + - + - + - +
Rapamycin
+ - + - + - -+ -
Actin
42 kDa
